# Resveratrol exhibits a strong cytotoxic activity in cultured cells and has an antiviral action against polyomavirus: potential clinical use

**DOI:** 10.1186/1756-9966-28-96

**Published:** 2009-07-01

**Authors:** Valerio Berardi, Francesca Ricci, Mauro Castelli, Gaspare Galati, Gianfranco Risuleo

**Affiliations:** 1 Dipartimento di Genetica e Biologia Molecolare, Sapienza Università di Roma, Roma, Italy; 2 Regina Elena Cancer Institute, Roma, Italy; 3 Dipartimento di Chirurgia "Pietro Valdoni", Roma, Italy

## Abstract

**Background:**

Resveratrol is a non flavonoid polyphenol compound present in many plants and fruits and, at especially high concentrations, in the grape berries of *Vitis vinifera*. This compound has a strong bioactivity and its cytoprotective action has been demonstrated, however at high concentrations the drug exhibits also an effective anti-proliferative action. We recently showed its ability to abolish the effects of oxidative stress in cultured cells. In this work we assayed the bioactivity of resveratrol as antiproliferative and antiviral drug in cultured fibroblasts. Studies by other Authors showed that this natural compound inhibits the proliferation of different viruses such as herpes simplex, varicella-zoster and influenza A. The results presented here show an evident toxic activity of the drug at high concentrations, on the other hand at sub-cytotoxic concentrations, resveratrol can effectively inhibit the synthesis of polyomavirus DNA. A possible interpretation is that, due to the damage caused by resveratrol to the plasma membrane, the transfer of the virus from the endoplasmic reticulum to the nucleus, may be hindered thus inhibiting the production of viral DNA.

**Methods:**

The mouse fibroblast line 3T6 and the human tumor line HL60 were used throughout the work. Cell viability and vital cell count were assessed respectively, by the MTT assay and Trypan Blue staining. Cytotoxic properties and evaluation of viral DNA production by agarose gel electrophoresis were performed according to standard protocols.

**Results:**

Our results show a clear dose dependent both cytotoxic and antiviral effect of resveratrol respectively at high and low concentrations. The cytotoxic action is exerted towards a stabilized cell-line (3T6) as well as a tumor-line (HL60). Furthermore the antiviral action is evident after the phase of virion entry, therefore data suggest that the drug acts during the synthesis of the viral progeny DNA.

**Conclusion:**

Resveratrol is cytotoxic and inhibits, in a dose dependent fashion, the synthesis of polyomavirus DNA in the infected cell. Furthermore, this inhibition is observed at non cytotoxic concentrations of the drug. Our data imply that cyto-toxicity may be attributed to the membrane damage caused by the drug and that the transfer of polyomavirus from the endoplasmic reticulum to the cytoplasm may be hindered. In conclusion, the cytotoxic and antiviral properties of resveratrol make it a potential candidate for the clinical control of proliferative as well as viral pathologies.

## Background

Murine polyomavirus (Py) is an ideal model system to investigate many different biological phenomena at cellular and molecular level. Polyomavirus is totally dependent on the metabolism of the infected cell: therefore, it has been used to study cellular and molecular functions. Classical works based on the study of the viral proliferation helped to elucidate the mechanisms of the regulation of DNA replication, RNA transcription and translation as well as tumor transformation. Analogously to other polyomaviruses, with which it shares a high sequence homology, Py can very efficiently transform non permissive cells in culture and is able to cause tumors if injected in immuno-suppressed or singeneic animals (see: [1] for a compendium on polyomaviruses and [2-7] for more recent reviews on this subject).

In last decade we investigated the role of both natural and synthetic substances on Py DNA replication and RNA transcription [8-10]. Also, the cellular and metabolic response after exposure to these substances was studied [11-15]. We particularly focused our attention on a natural complex mixture, known as MEX, obtained by methanolic extraction of whole neem oil [13]. This oil is prepared from the seeds of *Azadirachta indica *and has been extensively used in Ayurveda, Unani and Homoeopathic medicine possibly for centuries [16,17]. In our laboratory MEX showed a significant and differential cytotoxic action, with the cancer cells being more sensitive than the normal ones [18]. The main target of MEX is the plasma membrane which, after treatment with this extract, becomes more fluid without a substantial loss of its structural properties [19]. In addition, preliminary experiments performed in our laboratory suggest that MEX has also an antiviral activity (Berardi *et al*., in preparation); in any case a similar activity of neem leaf extracts was reported in a model of Dengue virus [20].

In this work we assayed the action of resveratrol (RV), a natural compound raising an increasing interest on the proliferation of cultured cells *i.e*.: the murine fibroblast line 3T6 as well as in the tumor line HL60. In addition, we also investigated the action of this drug on the proliferation of the murine polyomavirus in the infected cell population.

Resveratrol is a non-flavonoid polyphenol compound present in many plants and fruits, at especially high concentrations in the grape berries of *Vitis vinifera *[21]. This compound has a high bioactivity and its cytoprotective action has been demonstrated. As a matter of fact, possibly due to its polyphenol characteristics, RV was also shown to have antiviral action *versus *influenza A [22] and varicella zoster virus in cultured cells [23]. Analogous properties of RV against Herpes virus simplex I were shown in animal models [24]. In this latter case, suppression of transcription factor NF-κ-B seems to be involved in its antiviral property [25].

The results presented here show that RV exhibits a cytotoxic activity and has an antiviral property since it efficiently inhibits the synthesis of Py DNA. The inhibition is observed at non cyto-toxic concentrations of RV as shown by vital cell count and quantitative evaluation of the viral DNA synthesis after exposure to the drug. In addition, our results evidence a clear dose dependent antiviral effect of resveratrol. Since this action appears after the phase of virion penetration, data suggest that resveratrol exerts its antiviral properties during the synthesis of the viral DNA progeny. However because of its cytotxic properties, it may be envisaged an application of RV to control negatively the cell growth in proliferative diseases.

## Methods

### Cell cultures

The mouse fibroblast line 3T6 and the tumor line HL60 were used throughout the work. Cells were grown in high glucose DMEM, supplemented with newborn bovine serum (10% final concentration) glutamine (50 mM) and penicillin-streptomycin (10000 U/mL). Growth temperature was 37°C in controlled humidity at 5% CO_2_. Cells were routinely split and sub-cultured every third day.

Viral infection was performed at 4 pfu × cell^-1^, for 2 hours at 37° with occasional rocking. Infection procedure and extraction (replication assays) of *de novo *synthesized DNA were described in detail in previous works, see for instance [9,10,26]. Viral DNA was visualized after agarose gel electrophoresis in the presence of ethidium bromide (0.5 μg/ml, final concentration). Evaluation of cell vitality: Cell viability was assessed by the colorimetric MTT assay [27]. Absorbance was measured at 570 nm to obtain a standard cell count. The number of cells surviving to the treatment with RV (20 μM) was also evaluated by vital cell count in Trypan Blue in a Burker chamber. The same approach was adopted to count the cell mortality consequent to Py infection [18].

All experiments were repeated at least three times. The error bars indicate the Standard Deviation of the Mean (± SEM).

## Results

### Evaluation of the cytotoxicity of resveratrol and of the cell death consequent to Py infection

In preliminary experiments we assessed the concentration at which RV may exert a putative cytotoxic activity. It should pointed out, as a matter of fact, that natural substances endowed of cytoprotective and antioxidant properties, may present a threshold effect above which they can paradoxically show cytotoxic properties. The phenomenon has been documented for RV and its analogues as well as for curcumin another potent antioxidant drug with cytoprotective features [28-31].

**Figure 1:**
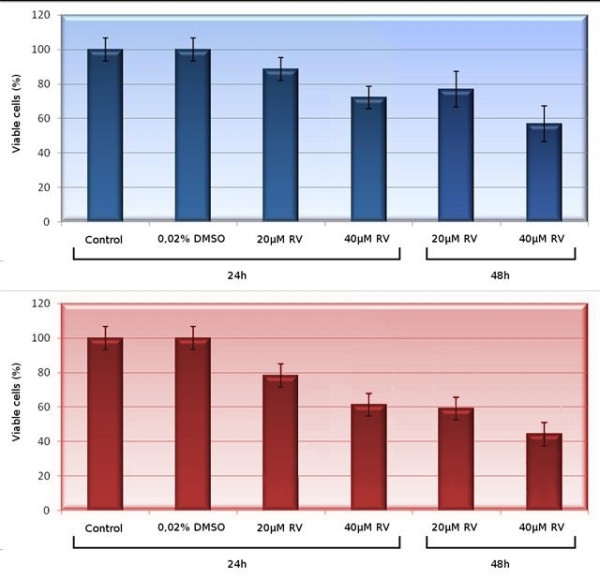
**Cytotoxicity of resveratrol assessed by the Mossman assay**. The bars report the percentage of viable cells after different times of exposure to the drug (24 hours: four bars to the left; 48 hours two bars to the right). The untreated control and the sample in DMSO at 48 hour are omitted since the data are virtually identical to the ones obtained at 24 hours. Data reported in upper panel refer to 3T6 cells while those shown in the lower panel refer to HL60 cells.

The cytotoxicity of RV on 3T6 cells has been evaluated by the Mossman assay [27] after treatment for 24 and 48 hours (Figure 1A and 1B, respectively), but in this latter case the treatment with 2 μM RV was omitted since this at this concentration the drug does not have a significant effect on cell mortality. The drug is dissolved in 0.02% DMSO (final concentration) in PBS but, at this low concentration, the organic solvent has no effects on cell survival, as shown by the second bar from the left. However cells exposed to RV at the concentrations of 20 and 40 μM show some sensitivity to the drug, since only about 60% of the cell population survives the treatment for 48 hours at the higher concentration. The vital cell count observed after trypan blue staining is in good agreement with the one obtained by the Mossman assay (Table 1, only the data for 20 μM RV at after 24 hour of treatment are shown).

**Table 1:** Vital cell count after trypan blue staining of cells treated with resveratrol (20 μM) for 24 hours.

**RV – Treated 3T6 Cells**		
Sample	Vital	Non-Vital

1	27	3
2	32	2
3	28	3
4	30	3
Average cell mortality 8.7 ± 2.6%		

We also investigated the cytotoxic activity of RV on the tumor cells HL60: a human promyelocytic leukemia cell line. The results clarly show that RV can significantly inhibit the cell growth already at a concentration of 25 μM.

Subsequently we assessed the level of cell mortality induced by Py infection: in this case we used the method of vital cell staining only with trypan blue. As a matter of fact, the MTT assay is informative of cell death deriving from membrane damage and former data from our laboratory indicated that the plasma membrane is actually one of the targets of RV. On the contrary, trypan blue staining has a more general action ranging from a generic damage of cell membrane to severe problems in cell homeostasis. Table 2 reports the vital cell counts in control and Py infected cells.

**Table 2:** Assessment of the cell mortality rate due to Py proliferation.

**Virus Py 24 h**		
**Sample**	**Vital**	**Non-Vital**

**1**	44	1
**2**	45	2
**3**	41	2
**4**	52	1
Average cell mortality 3,4% ± 1,5%		

**Virus Py 48 h**		

**Sample**	**Vital**	**Non-Vital**

**1**	40	2
**2**	46	4
**3**	44	4
**4**	49	2
Average cell mortality 6,7% ± 2,5%		

The vital cell count was evalutated by trypan blue staining.

The reported data show that after 48 hours of infection the cell death rate is about as double as in controls: however the viral infection does not seem to cause extensive loss cell vitality.

In the light of these results the effect of RV on Py proliferation was evaluated at 24 hours post-infection in cells were treated with 20 μM RV or at the concentrations of drug reported in the legends to the figures.

### Effect of resveratrol on the viral proliferation

**Figure 2:**
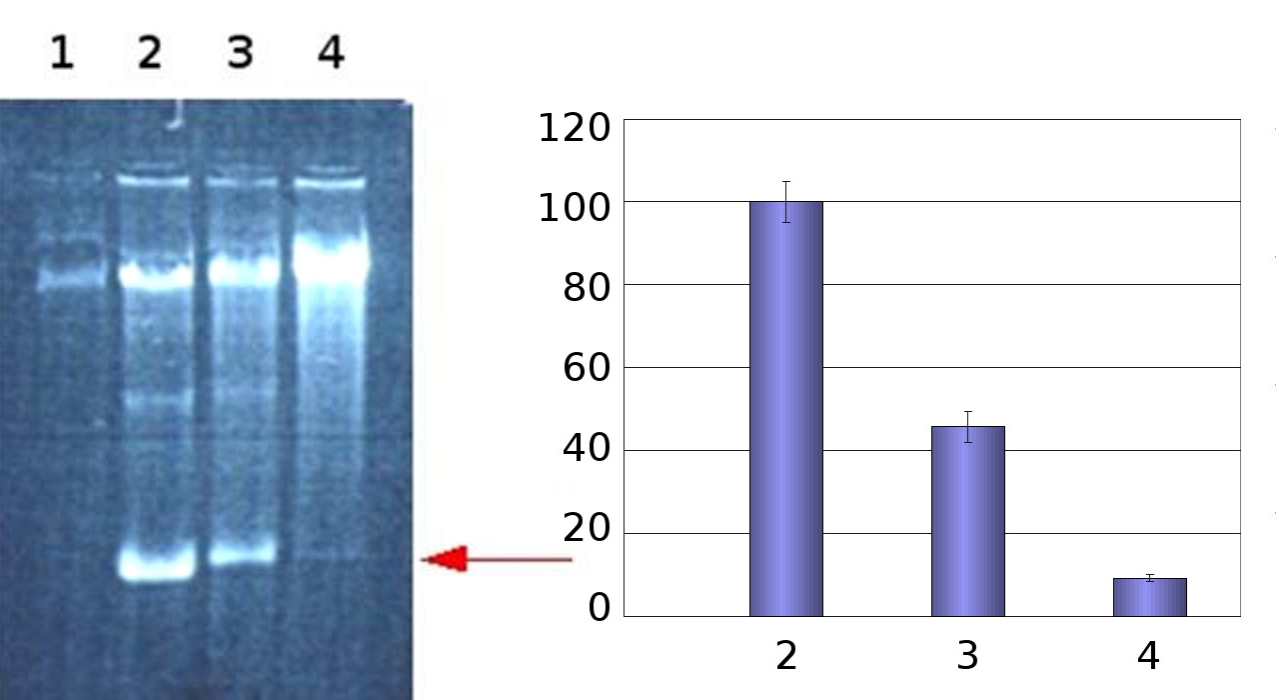
**Viral DNA yield obtained at 24 hours post-infection**. Left panel: Electropherogram of the *de *novo synthesized progeny viral DNA (form I) indicated by the arrow. Lane 1: Mock infected cells, Lane 2: Untreated control cells; Lane 3 and 4: Cells treated with RV 20 μM and 40, respectively. Right panel: Quantification of the fluorescence bands reported in the left panel. The yield of the viral DNA is normalized to the amount obtained in untreated control cells (Bar 1). Bar 3 and bar 4: viral DNA obtained after treatment with RV 20 μM and 40, respectively

Semi-confluent cells were infected with Py and RV was added after the absorption phase at the indicated final concentrations. Infection was continued for 24 hours and progeny viral DNA was extracted according to the Hirt-procedure [26] (Figure 2A). The data clearly show that the viral replication is virtually abrogated at 20 μM RV. Infections performed in the presence of RV during the absorption phase gave essentially the same results (not shown).

**Figure 3:**
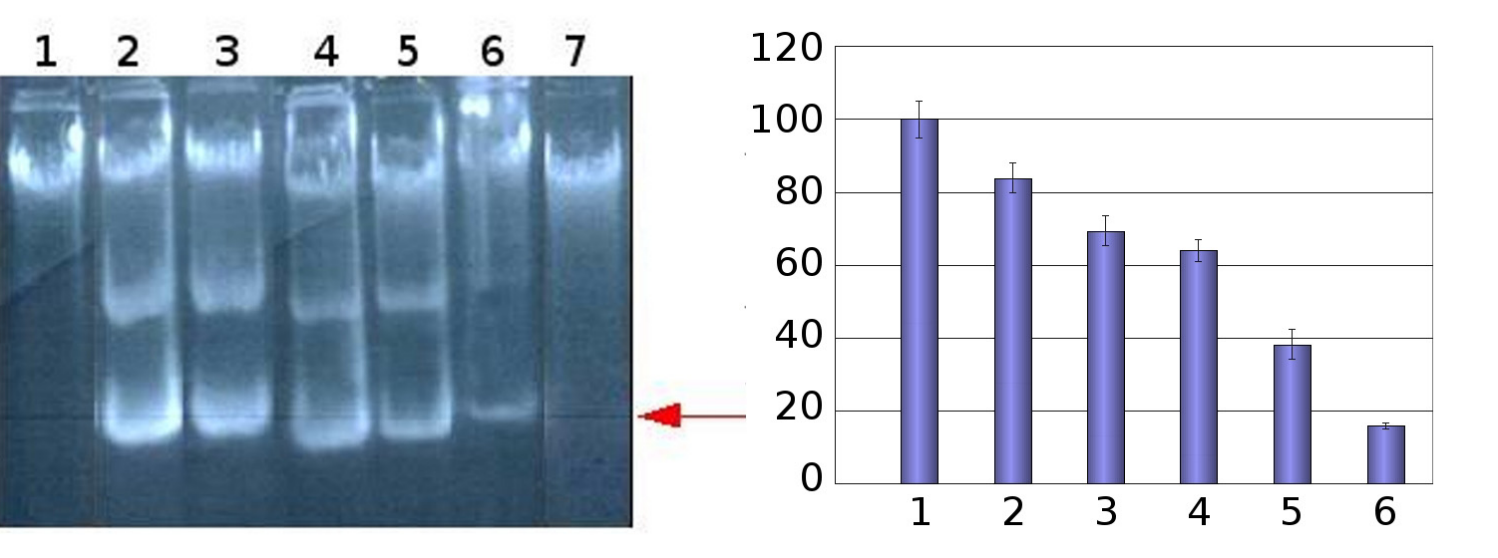
**Decrease of viral DNA as a function of the duration of the exposure to resveratrol**. Left panel: Progeny viral DNA (form I) is indicated by the arrow. In this case, the culture medium was changed to fresh drug-free medium at the following times post-infection. The incubation was continued for 24 hours. Lane 1: Mock infected cells; Lane 2: Untreated control cells; Lane 3 through 6: 4, 8, 12 and 16 hours, respectively; Lane 7: The medium was not changed and infection was carried permanently in the presence of RV (20 μM). Right panel: Quantification of the fluorescence bands reported in the left panel. The yield of the viral DNA is normalized to the amount obtained in untreated control cells (Bar 1). Withdrawal of RV is reported in the legend to left panel of this figure.

To assess whether the continuous presence of RV is necessary to inhibit the viral replication we removed the drug at different time points after the viral penetration into the cell (Figure 3). Therefore, the infection was carried out in 20 μM RV but the culture medium was changed to a drug-free fresh medium after different times of treatment and the incubation was continued for 24 hours. Results show that removal of RV after four hour incubation has little or no effect on the yield of viral progeny DNA (lane 2). The drug must be present for the whole infection time to be effective and to cause the complete inhibition of the viral replication (lanes 6 and 7).

## Discussion

In this work we report on cytotxicity *versus *two different cell lines: a normal mouse firbroblast line and tumoral one. The results clearly show that RV can exert a cytotoxic action both against a normal stabilized fibroblast cell line and human tumor cells. The human tumor line seems to be slightly more sensitive to the drug and this recalls results previously obtained in our laboratory with MEX: a partially purified natural mixture [18]. The antiviral activity of resveratrol towards murine polyomavirus infection was also evaluated. The exposure to the drug was carried at a concentration of RV which did not show a significant cytotoxic effect. It is known that resveratrol can exert anti-oxidant and anti-inflammatory activities and, also, it regulates multiple cellular events associated with carcinogenesis: for a relatively recent review see [28]. The cytotoxicity of RV is only apparently paradoxical; as a matter of fact this drug induces cell cycle arrest and stimulates the Reactive Oxygen Species (ROS) activated mitochondrial pathway leading to apoptosis [29,30]. An analogous paradoxical action has been described for another potent antioxidant: curcumin, which is able to induce apoptosis in human cervical cancer cells [31]. Therefore, an evaluation of possible cytotoxic effects of RV in our model system was a necessary pre-requisite. Murine polyomavirus productive cycle ends with the lysis of the infected cell: hence the actual number of cells dying as a consequence of viral proliferation had to be also assessed. The results of these experiments allowed us to find out the best conditions where the putative antiviral activity on murine Py could be investigated.

The results presented in this work show that like in the case of influenza A, HVS and varicella-zoster [22-25], the viral replication is severely inhibited by RV also in the case of murine Py. The inhibition is dose and time dependent and all experiments were carried out at 24 hour of infection time when the effects on the cell viability due to the exposure to the drug or to the viral proliferation are minimal. Similar results were obtained after 42 hours of infection but after such a prolonged time the significant cell mortality induced by RV and by the progression of the viral infection could overlap and/or mask the actual effects attributable to the drug (infection data non shown). Furthermore infection experiments performed in the presence of RV during the absorption phase gave essentially the same results obtained in infections experiments where drug was added after the viral penetration (not shown). This strongly suggests that virus entry is not the main target of RV whose action is therefore exerted during the phase of viral DNA synthesis. Furthermore, the presence of the drug for the whole duration of the infection is necessary to abrogate completely the viral DNA production. As a mater of fact exposure to the drug for shorter time has no effect on the overall yield of viral DNA. Incidentally, this data also shows that the intracellular "life time" of the viral DNA is fairly long, since removal of the drug after 12 hours exposure seems to have little effect on the amount of the progeny DNA. These data recall a similar observation made in our laboratory with a different natural substance [9].

At the moment the mechanism of action of RV remains to be elucidated; however in the case of influenza A virus, the translocation of viral ribo-nucleoprotein complexes to the endoplasmic reticulum is hindered and the expression of late viral proteins is reduced. These two phenomena could be related to the inhibition of protein kinase C activity and its dependent pathways [22]. Also, Py utilizes protein-protein complexes associated to the endoplasmic reticulum and involving the viral capsid proteins VP2 and VP3 [32]. Therefore it could be speculated that the viral transfer to the nucleus, in the case of Py, follows an analogous process.

## Conclusion

The results presented in this work demonstrate a clear, dose dependent cytotoxic and antiviral effect of resveratrol: cytotoxicity at high concentration of the drug both on normal and tumor cells. On the other hand at low concentration, the continuous presence in the culture medium is necessary for the drug to be effective. The target of RV is the replication of viral DNA; however further studies are required for the full elucidation of the inhibitory mechanism mediated by RV leading to the abrogation of the viral DNA synthesis. This effect was demonstrated in the absence of significant cytotoxic effects induced by the drug. Removal of RV at short time after infection does not have a significant effect on the production of viral progeny DNA and this suggests that the viral penetration is not the main target of the drug. Therefore we may conclude that the RV dependent inhibition of the viral proliferation occurs at subsequent stages: possibly during translocation of the virion from cytoplasm to nucleus.

Finally this work gives a further support to the possibility that RV may find a potential clinical use for the control of proliferative pathologies and/or as an antiviral drug.

## Competing interests

Authors declare that no conflicting or competing interests, of any nature, exist between the Authors of this work and their Academic activity.

## Authors' contributions

All Authors equally contributed to the completion of this work.
